# Olfactory Dysfunction in Patients with Chronic Rhinosinusitis

**DOI:** 10.1155/2012/327206

**Published:** 2012-05-20

**Authors:** María V. Sánchez-Vallecillo, María E. Fraire, Carlos Baena-Cagnani, Mario E. Zernotti

**Affiliations:** Otolaryngology Department, Sanatorio Allende, Independencia 757 3rd Floor, Nueva Córdoba. Córdoba CP 5000, Argentina

## Abstract

*Objectives*. To measure the prevalence of and identify the clinical characteristics associated with olfactory decline in patients with chronic rhinosinusitis. *Methods and Materials*. There is analytical, prospective, and observational study in adult patients with a diagnosis of chronic rhinosinusitis. The olfactory test used was the Connecticut Chemosensory Clinical Research Center (CCCRC). *Results*. They are 33 patients total. Within the group of patients aged 18 to 39, 9% had normosmia, 73% hyposmia, and 18% anosmia (*P* < 0.001). Between 40 and 64 years old, there was no patient with normosmia, 63% hyposmia, and 37% anosmia (*P* < 0.001). Of patients older than 65 years old, 33% showed mild hyposmia, 34% severe hyposmia, and 33% anosmia (*P* < 0.001). 52% were females, and 48% were males. *Conclusion*. Nasal polyposis, asthma, septal deviation, turbinate hypertrophy, tobacco, and allergic rhinitis are predicting factors of olfactory dysfunction. Antecedents of previous endoscopic surgeries, age, and gender would not be associated with olfactory loss.

## 1. Introduction

 Between 14 and 30% of those patients who suffer from chronic rhinosinusitis have olfactory dysfunction, a condition that affects more than 10 million people [1]. Inflammatory conditions such as rhinosinusitis are characterized by having two components: the inflammatory component itself and the conductive component [2], which impedes the arrival of odorants to the olfactory epithelium [3]. Mott and Leopold [2] explain that the function of the neuroepithelium can be damaged by a local inflammation which impedes the arrival of odorants to the receptors of cilia. According to them, this is caused by an edema of the neuroepithelium that produces the stretching of the primary olfactory neuron and impedes, in this way, the transmission of synaptic impulses, and by the destruction or damage of olfactory receptors caused by the products of inflammation. Despite the advances to understand the mechanisms of olfactory dysfunction, its clinical treatment is still limited. Some studies show that olfactory loss is associated with factors such as age, gender, and exposure to toxic agents and tobacco [3]. However, the relationship between olfactory dysfunction and chronic rhinosinusitis has been rarely studied. Certain researchers have suggested that the causes of olfactory dysfunction are nasal polyposis, allergic rhinitis, asthma, septal deviation, turbinate hypertrophy, and rhinosinusal surgery, but an agreement has not been reached yet [3, 4]. On the other hand, some studies have shown that olfactory levels are variable after medical and surgical treatment of chronic rhinosinusitis [4]. In this observational prospective analytical study, the clinical characteristics and the prevalence of olfactory dysfunction in patients with chronic rhinosinusitis will be analyzed. The results of this study will help ENT doctors to take into account different factors that may cause olfactory loss and, in this way, give a more objective treatment.

## 2. Methods and Materials

 33 adult patients (greater than or equal to 18 years old) with a diagnosis of chronic rhinosinusitis whose medical treatment has been insufficient up to now and who were candidates to undergo endoscopic nasal surgery were studied. To confirm the diagnosis of chronic rhinosinusitis, a physical examination and a CAT scan based on the system of staging of chronic rhinosinusitis proposed by Lund-Mackay were done to patients. This prospective study was carried out from May to October 2010. The following criteria to select patients were taking into account: age, gender, tobacco consumption, previous rhinosinusal surgeries, nasal polyposis, asthma, Lund-Mackay staging greater than or equal to 11, allergic rhinitis confirmed by prick test (according to a report made by an allergy doctor.) Patients under 18 years old, immunodeficient, with an autoimmune disease or with cystic fibrosis were excluded from the study. 

## 3. Olfactory Function Measurement

 The olfactory test used in this study is the Connecticut Chemosensory Clinical Research Center (CCCRC), in which a threshold test, an identification of odors, and a trigeminal evaluation were carried out [5].

 This first examination called *Threshold test or Luminary test* consists of using a stock solution with 4% butanol and then diluting it with distilled water progressively to one third, until you complete eight dilutions in eight different flasks. The flask with 4% butanol is number 0, the most concentrated, and the most diluted is number 8. The test starts presenting the patient with flask number 8 and another with distilled water. It is brought closer to the left naris first, and the patient is asked to cover the right naris; movements in each flask have been made to make the vapor with odorant molecules reach the top. The patient smells one flask (with butanol), and then the other (with distilled water) or vice versa, and as the method is of forced choice to avoid biased answers, we ask the patient to indicate which flask has the stimulus, without identifying it. When the flask with butanol is chosen 5 times in a row, without mistaking, that is, the threshold. If there is a mistake, the patient passes to the following dilution. Then the same is done in the right naris. The reference values are as follows:

normosmia: flasks 6 and 7
mild hyposmia: flask 5
moderate hyposmia: flask 4
severe hyposmia: flasks 2 and 3
anosmia: flasks 1 and 0.

As in the previous method, the second examination called *smell identification test or supraliminal test* also consists of making the patient smell through both nares separately having a list with the names of odorants shown and other distracters. The odorants used are coffee, chocolate, vanilla, talc, soap, oregano, and naphthalene. The values of the results are the following:

normosmia: identification of 6 or 7 stimuli
mild hyposmia: identification of 5 stimuli
moderate hyposmia: identification of 4 stimuli
severe hyposmia: identification of 2 or 3 stimuli
anosmia: identification of 1 or none stimulus.

*The arithmetic mean is calculated between the value of the Threshold test and the Smell Identification test, which is the total or compound scoring *(Table 1) [5].

 Finally, an *evaluation of the trigeminal component* is done: with 4% butanol.

## 4. Statistical Analysis

 Data was analyzed using GraphPad Prism 4, statistical software. The prevalence of normosmia, hyposmia, and anosmia was calculated taking into account three age groups: 18–39, 40–64, and more than 65 years old.

 To calculate the differences between the three olfactory categories considering age and gender, the two-way ANOVA test and the Bonferroni posttest were used. To determine the relationship between hyposmia or anosmia and the clinical characteristics present in patients, the two-way ANOVA test and the Bonferroni post-test were used. 

## 5. Results

**Figure 1:**
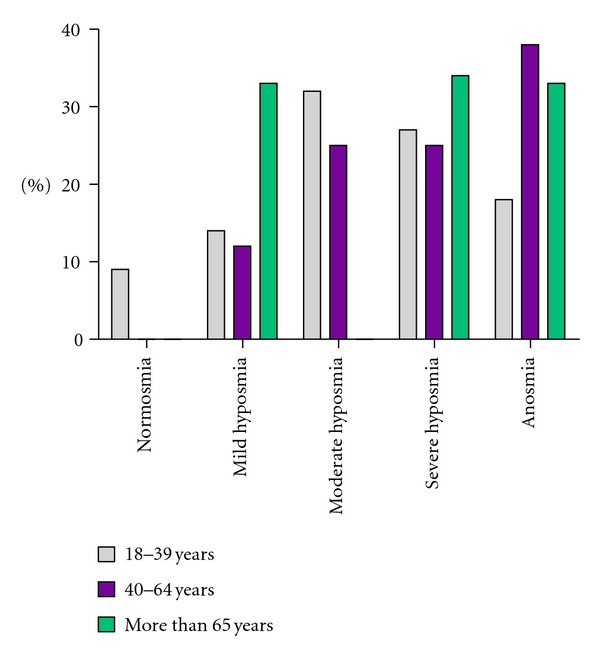
Age-related olfactory alteration.

 Out of a total of 33 patients, it was observed that within the age range of 18 to 39 years old, 9% showed normosmia, 73% hyposmia, and 18% anosmia (*P* < 0.001). Within the range of 40 to 64 years old, no patient with normosmia was registered, whereas 63% had hyposmia and 37% anosmia (*P* < 0.001). In the case of those patients older than 65 years old, 33% showed mild hyposmia, 34% severe hyposmia, and 33% anosmia (*P* < 0.001) (Figure 1). 

**Figure 2:**
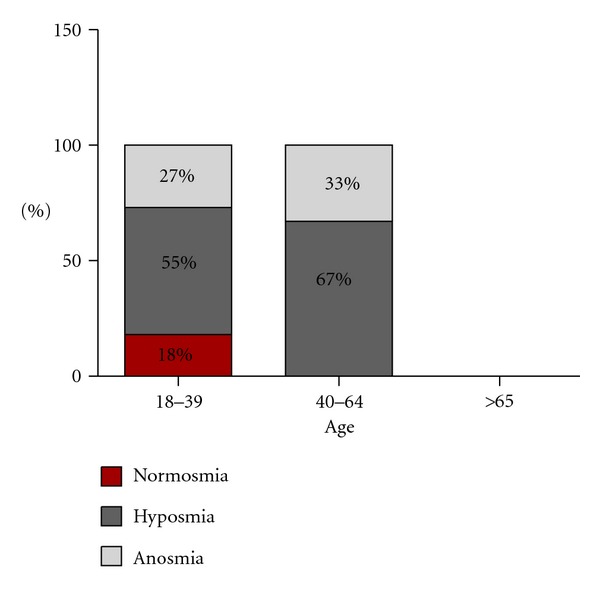
Female patients.

 The percentage of female patients was 52%. In the age range between 18 and 39 years old 18% showed normosmia, 55% hyposmia, and 27% anosmia; between 40 and 64 years old, 67% had hyposmia and 33% anosmia; there were no female patients registered older than 65 years old (Figure 2).

**Figure 3:**
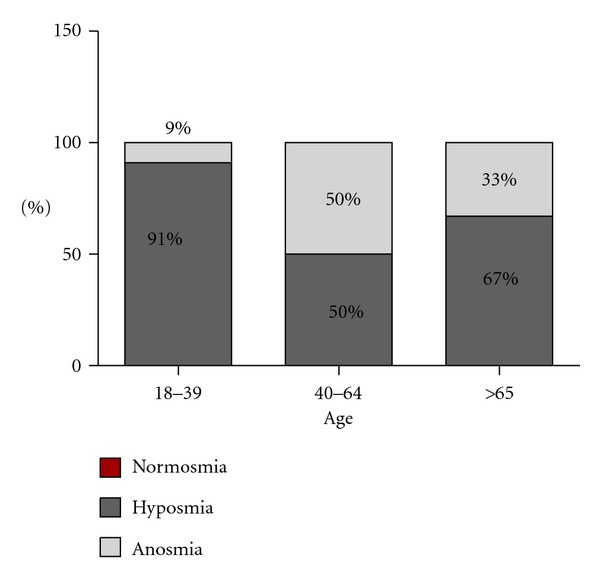
Male patients.

 In the case of male patients, which were 48%, within the group of 18 to 39 years old, it was registered that 91% of the patients suffered from hyposmia, and 9% from anosmia; between 40 and 64 years old, 50% showed hyposmia, and 50% anosmia; in patients older than 65 years old, 67% presented hyposmia, and 33% anosmia. As it can be observed, no patients with normosmia were registered (Figure 3).

**Figure 4:**
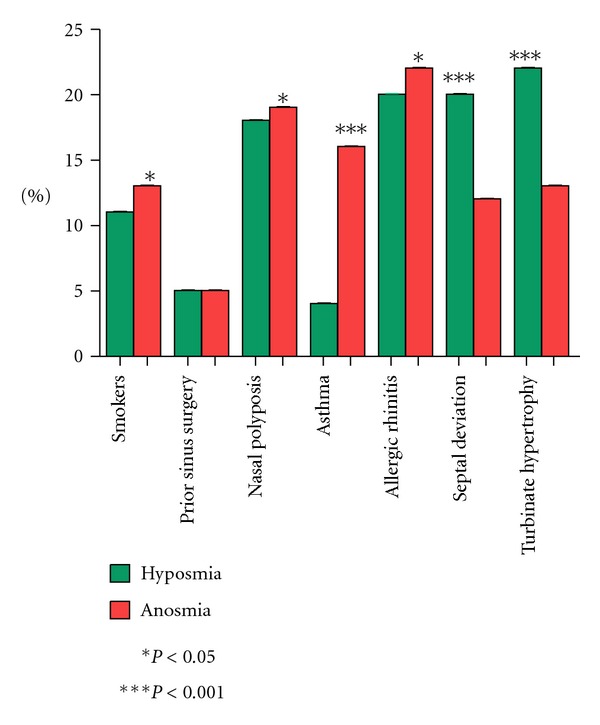
Clinical characteristics associated with olfactory dysfunction.

 A relationship between hyposmia and anosmia with the clinical characteristics of each patient was established. Those participants who showed 2 or more of the same conditions were counted as individual cases. 72% suffered from hyposmia and 28% from anosmia, of which 11% of hyposmic patients and 13% of anosmics were tobacco consumers (*P* < 0.05); 5% in both cases had antecedents of nasal endoscopic surgery. Nasal polyposis was shown in 18% of hyposmic patients and in 19% of anosmics (*P* < 0.05). As regards asthma, there was a significant difference in patients with hyposmia that represented 4%, unlike those who suffered anosmia that were 16% (*P* < 0.001). In the case of allergic rhinitis, 20% suffered from hyposmia while 22% from anosmia (*P* < 0.05). Septal deviation was present in 20% of hyposmics, but significantly greater in anosmics who were 12% (*P* < 0.001). Finally, it is observed that turbinate hypertrophy in hyposmia is 22%, a significant difference compared with anosmic patients who were 13% (*P* < 0.001) (Figure 4). It is worth mentioning that in the obtained results of clinical characteristics, normosmic patients were excluded. 

## 6. Discussion

 From the analysis of clinical characteristics, included in this study, in relation with olfactory decline, it can be observed that nasal polyposis, septal deviation, and turbinate hypertrophy are associated with olfactory dysfunction in patients with chronic rhinosinusitis, and, to a lesser extent, with tobacco and allergic rhinitis. It is important to highlight that age, gender, and antecedents of endoscopic surgery are not significantly associated with olfactory loss. Moreover, no evidence of interaction or effect between gender and chronic rhinosinusitis was found.

 Evidently, in this study as well as in literature, the relationship between chronic rhinosinusitis and olfactory dysfunction seems to be multifactorial and complex. It was thought that rhinosinusitis consisted of a disorder of conduction caused by an olfactory tract mechanical obstruction produced by an edema, secretions, or nasal polyposis. However, there are other causes that contribute to olfactory dysfunction [4]. Kern [6] could observe that patients with chronic rhinosinusitis have an evidence of direct inflammation on the neuroepithelium; the severity of olfactory loss depends on the degree of these inflammatory changes. This helps to understand the behavior of systemic corticoids, which allows us to define that in the olfactory dysfunction intervenes a conductive and neurosensory process. Despite the advances in the clinical handling of patients with rhinosinusitis and olfactory loss, the treatment with corticoids and surgery has been demonstrated to have variable results.

 According to this study, age would not be a risk factor for olfactory loss, unlike the ideas proposed by Doty and Mishra [4]. Patients with chronic rhinosinusitis and anosmia show a moderate-to-severe change in the mucus, which inhibits, in this way, olfactory neurogenesis. As a consequence, one of the questions is to know if rhinosinusitis and age would have a synergic effect in the condition of olfactory epithelium in older patients [7].

 Tobacco would be a factor associated with olfactory loss. Other studies have demonstrated that olfactory function is conversely related to the cumulative dose of tobacco [8].

 In this study, nasal polyposis is associated with hyposmia and anosmia. Patients with nasal polyposis suffer a conductive olfactory loss, caused by a physical obstruction in the airways; for this reason, degenerative changes associated with recurrent infections and chronically used nasal medication may be experienced. These results coincide with other studies. Perry and Kountakis [9] reported high olfactory dysfunction results in patients with chronic rhinosinusitis and nasal polyposis compared with patients that did not suffer from polyps. Vento et al. [10] found that 46% of the patients with nasal polyposis had a greater index of olfactory loss unlike those patients of the same age without polyps.

 In the case of asthma, it is observed that 20% of the patients suffered from anosmia; it can be thought that those patients with asthma and chronic rhinosinusitis have a systemic inflammatory response in the high and low respiratory tract. This inflammatory process would cause olfactory loss [11].

 Allergic rhinitis would be another factor that contributes to the olfactory decline, according to the values obtained in the studied group. Apter et al. [12] affirms that patients with allergic rhinitis have greater risk of suffering from an olfactory loss as a result of recurrent infections in the respiratory tract, which causes a great damage to the neuroepithelium. In contrast to Simola and Malmberg [13] who got patients with allergic rhinitis with a greater degree of olfactory loss compared with patients who suffered from perennial allergic rhinitis.

 Antecedents of previous sinus surgery are not associated with olfactory dysfunction. It was thought that various mechanisms would cause an olfactory lesion after a surgery, for example, a direct lesion mechanism against the olfactory epithelium, an airway change, the effects of pharmacological agents, or vascular lesions, and ischemia. 1% anosmia after a sinus surgery has been reported [14, 15]. However, there was no increase in the risk of olfactory loss in those patients that previously underwent a nasal endoscopic surgery compared with those who did not. Previous studies [5, 13] underline that a history of nasal polyposis surgeries is associated with olfactory function loss, but it is not that way in patients with antecedents of chronic rhinosinusitis surgeries.

 Septal deviation and turbinate hypertrophy cause olfactory dysfunction due to a physical obstruction in the nasal airways. Different studies show variable results as regards olfactory function after a surgical treatment due to turbinate hypertrophy or septal deviation. In the case of Kimmelman [14] there is no statistical significance in patients who underwent septoplasty. Conversely, Damm et al. [16] show that about 80% of patients improved odor identification after the surgery. Septoplasty and turbinectomy can relieve nasal obstruction and, in this way, get a better olfactory function.

## 7. Conclusion

 Olfactory dysfunction is common in patients with chronic rhinosinusitis. Nasal polyposis, asthma, septal deviation, turbinate hypertrophy, tobacco, and allergic rhinitis are predicting factors of olfactory dysfunction. Antecedents of previous endoscopic surgeries, age, and gender would not take part in olfactory loss. These findings would, in the future, help to get a better understanding about the mechanisms that produce olfactory dysfunction in patients with chronic rhinosinusitis.

## Figures and Tables

**Table 1:** CCCRC test scoring.

6.0–7.0	Normosmia
5.0–5.75	Mild hyposmia
4.0–4.75	Moderate hyposmia
2.0–2.75	Severe hyposmia
0–1.75	Anosmia
